# Voice and Choice: Voice Equivalence and Representational Choice in Virtual Health Assistants for Colorectal Cancer Screening Among Black American Adults

**DOI:** 10.1145/3717511.3747065

**Published:** 2025-10-08

**Authors:** Andrew Maxim, Eric Cooks, Janice L. Krieger, Benjamin Lok

**Affiliations:** Computer & Information Science & Engineering, University of Florida, Gainesville, FL, USA; The Leukemia & Lymphoma Society, New York, NY, USA; Mayo Clinic Comprehensive Cancer Center-Florida, Jacksonville, FL, USA; Computer & Information Science & Engineering, University of Florida, Gainesville, FL, USA

**Keywords:** virtual humans, healthcare, patient choice, computer-generated voice

## Abstract

Advances in AI, including large language models, enable virtual health assistants (VHAs) to tailor messaging to cultural and individual needs. However, to fully realize this potential, VHA voices must match or exceed human voices in influencing behavioral intentions. We conducted a multi-study investigation on whether matching text-to-speech (TTS) voices to human voices across vocal traits could promote colorectal cancer (CRC) screening among Black or African American adults. Study 1 (N = 313) tested whether a TTS voice prosodically matched to a human voice could elicit equivalent screening intentions using a White male VHA. It also evaluated whether increasing vocal confidence via SSML would enhance effectiveness. A user preference study (N = 317) assessed VHA identity and delivery modality preferences, finding strong support for a Black female VHA. Building on these results, Study 2 (N = 106) replicated the TTS-human voice comparison using a Black female VHA. Across studies, matching TTS voices to human voices on frequency, speech rate, and pitch variation achieved equivalent screening intentions. We discuss implications for VHA design and user-centered voice adaptation.

## 1 Introduction

Virtual humans have become increasingly prominent across various fields, from entertainment and education to healthcare. A specialized role of virtual humans, virtual health assistants (VHAs) are tailored specifically for medical and wellness applications. VHAs have been developed to examine their use in increasing patient disclosure [12], providing mental healthcare information [25, 42], and providing healthcare information and support [30].

VHAs offer many advantages in scalability, engagement, and adaptability. VHAs have a relatively low cost of production and can be accessed asynchronously by patients at the time and place of their choosing. However, it is the ability to dynamically alter a VHA-led healthcare intervention that gives designers the most impactful form of control[41]–allowing them to tailor content, tone, and delivery in real-time based on user traits, demographics, and responses. With the growing library of large language models (LLM)–and with the addition of fine-tuning and retrieval-augmented generation (RAG) to a specific context–dynamic, customized VHA message delivery is growing ever more prolific and producing better outcomes for participants [32]. However, an area of VHA design that remains a challenge to tailoring messaging is virtual human voice, where pre-recorded human voices continue to be strongly preferred.

Recorded human voices have traditionally been the standard for healthcare interventions led by VHAs, primarily due to their naturalness. However, reliance on human recordings limits the ability to dynamically tailor messages to better suit participant culture, demographics, and individual needs. In contrast, computer-generated speech, such as text-to-speech (TTS) synthesis, offers the potential for real-time adaptability, though its effectiveness has produced mixed results in prior VHA interventions[2, 9, 10, 16]. Thus, a clearer understanding is needed of how TTS voices can achieve outcomes equivalent to or better than human voices in healthcare contexts.

Addressing this gap, we conducted a multi-part study comprising two experimental studies and a user preference study. Our primary focus was to explore whether closely matching a TTS voice’s pitch, speech rate, and pitch variation to a human voice would influence participants’ intention to screen for colorectal cancer (CRC) within a VHA-led intervention. We selected CRC screening among Black or African American adults in the United States as the health context—an area where VHA interventions have shown promise[6, 21, 38] and where persistent disparities in CRC outcomes remain[36]. In particular, Black men experience a 44% higher mortality rate than White men, highlighting the urgent need for culturally responsive, adaptable interventions that TTS technology could enable. Understanding the specific preferences of Black or African American adults is essential for developing dynamic, adaptable interventions that are not only culturally responsive but also capable of personalizing engagement to improve CRC screening outcomes.

Accordingly, our research was guided by the following questions:

**RQ1:** How does closely matching a TTS voice’s pitch, rate of speech, and pitch variation to a human voice affect participant intention to screen for CRC in a VHA-led healthcare intervention?
**RQ2:** What are Black or African American adults’ preferences regarding VHA characteristics, modality, and physician involvement in a VHA-led CRC screening intervention?

In addressing RQ1 in Studies 1 and 2, we adopted an equivalence testing approach, where the alternative hypothesis specifies that outcomes between TTS and human voice conditions fall within a predefined equivalence margin[22, 26, 35]. Conversely, the null hypothesis posits that a significant difference exists. Separately, to further investigate how vocal characteristics influence participant intentions, Study 1 examined whether modifying TTS voices to convey greater expertise and vocal confidence would enhance behavioral intentions, following standard hypothesis testing procedures.

Thus, we formulated two hypotheses:

**H1:** Participants interacting with a VHA using a vocally matched TTS voice will have equivalent intentions to screen for CRC as those interacting with the human voice condition.
**H2:** Participants interacting with the VHA using a TTS voice altered to increase expertise and vocal confidence will have higher intentions to screen.

The findings from our work demonstrate that TTS voices can have an equivalent impact on behavioral intentions when closely matched to a human voice on key prosodical traits. While this finding does not provide an easy solution in initial voice selection, it paves the way towards replacing human voices with equivalent TTS voices. Further, we discuss preferences when provided a choice in VHA-led interventions among this underserved population, particularly VHA model race and gender. Lastly, this research contributes to the growing body of work on virtual human and virtual health assistant design and the effects on behavioral intention.

## 2 Related Work

### 2.1 Virtual Health Assistants for Healthcare Screenings

Virtual health assistants represent a transformative method for delivering healthcare interventions, particularly in increasing accessibility and patient autonomy. Prior research has shown VHAs can effectively support chronic disease management [4], psychological education [30], and mental health symptom reporting [24]. VHA-led interventions have also been used in colorectal cancer treatment [21, 39, 43], an area where stigma and discomfort often deter patients from engaging in routine screening.

While prior research establishes that VHAs can successfully deliver health content, many studies use static designs with little customization to the patient’s identity or preferences. Further, understanding how voice modality influences behavioral outcomes has been underexplored. In Study 1 of the present work, we investigate how three different voice types (a human voice, a TTS voice matched on pitch and vocal parameters, and an altered TTS voice designed to enhance vocal confidence and expertise) impact intentions to screen for CRC. This study uses a White male VHA to establish baseline effects of vocal presentation on behavioral intentions.

### 2.2 Virtual Human Voice Effectiveness

Many VHA-led interventions still rely on human voice recordings, which are costly, inflexible, and difficult to update. Several studies have examined differences between human and TTS voices with mixed results. Abdulrahman et al. found no significant differences between a human voice and a TTS voice using similar levels of tone or expressiveness on co-presence, trust, or working alliance[2]. In a similar study, Abdulrahman and Richards found no difference in likeability between a human and a TTS voice[1]. In contrast to this, studies by Craig et al. and Do et al. found virtual humans using TTS voices to be less trustworthy than human voices[8, 9]. However, in the case of the Do et al. study, there was a 79% difference in pitch and an 8% difference in the rate of speech between the study’s human and neural voices. Notably, these studies did not attempt to match prosodic features between voice types, and more broadly, there has been little research investigating whether TTS voices matched on prosody with human voices can produce comparable effects on health-related outcomes.

In Study 1, we compare the effects of a human voice, a closely matched TTS voice, and a speech synthesis markup language (SSML) modified TTS voice designed to express greater confidence and expertise. These manipulations directly test whether TTS voices can maintain persuasive effectiveness while offering the flexibility for scalable VHA-led interventions.

In Study 2, we extend this question using a Black female VHA based on participant preference findings from our user preference study. Participants were exposed to either a human or a matched TTS voice, allowing us to test whether the voice effects found in Study 1 replicate across different VHA model demographics.

### 2.3 Expertise and Confidence in Healthcare

In healthcare communication, credibility and expertise are foundational to trust. Patients assess not only what is said but also how confidently and credibly information is delivered. Research has shown that clarity, vocal authority, and message consistency all affect perceived healthcare credibility [29, 33]. These factors influence not just patient attitudes but also actual decision-making behavior.

The voice of medical professionals significantly influences how patients perceive trust, competence, and emotional connection. Prosodic traits – such as pitch, rate of speech (RoS), and pitch variation – have long been tied to the perception of personality traits and credibility [3, 20, 27, 34]. Specifically, confidence and expertise are often conveyed vocally through faster speech rates, lower pitch, and greater pitch variability [14, 18, 37].

Much of this work has focused on human providers and not on VHAs. However, few studies have examined how TTS voices – particularly those engineered to mimic credible vocal profiles – affect patient trust and behavioral intentions. Using SSML to manipulate vocal parameters in Study 1 offers a novel way to explore whether perceived vocal confidence and expertise can be achieved using TTS voices. By matching and altering TTS voices along key dimensions, we examine whether the perceived expertise and confidence of a VHA can be enhanced without sacrificing the adaptability offered by TTS voice systems.

### 2.4 Healthcare Intervention Preferences

Healthcare disparities remain a significant barrier to effective screening, especially for Black and African American patients, who face higher CRC mortality rates and often report lower trust in healthcare systems [36]. Cultural concordance – such as the ability to choose a provider who shares one’s race or gender – has been linked to increased comfort and trust in medical interactions [31]. Similarly, patients who feel they had a say in selecting their provider report higher trust and satisfaction [17].

While this has been examined in human healthcare settings, little research has explored how these preferences translate to virtual environments. However, no study, to our knowledge, has systematically tested whether allowing users to choose the race, gender, or modality of their virtual health assistant influences their engagement or perceptions. In our user preference study, we offered Black and African American participants aged 45–75 three points of choice in a CRC screening intervention: (1) the race/gender of the VHA (Black male, Black female, White male, White female), (2) whether the intervention was introduced by a race- and gender-matched human doctor, and (3) whether the intervention was delivered via a VHA or in text-only format.

We report on participants’ preferences for these options, which informed the design of Study 2, where the Black female VHA was used. This choice-based design directly tests how aligning VHA-led interventions with user identity preferences may enhance perceived relevance and trust.

## 3 Study 1: Investigating the Effects of Voice Modality

Our first study investigated how TTS voices closely matched to a human voice in three features of vocal prosody impact participant intentions to screen for CRC in a virtual-human-led healthcare intervention.

### 3.1 System Description

#### 3.1.1 Voice Conditions.

In Study 1, we employed three different voice conditions to study the effects of TTS voices on intentions to screen for CRC: human voice, matched TTS voice, and altered TTS voice conditions (see Section 3.2.1 for Study Design).

##### Human Voice Condition.

The recorded human voices were acquired from prior VHA studies on promoting colorectal cancer screening[38, 39, 43] and had been used successfully with thousands of participants[21].

##### Text-to-speech Voice Condition.

We used MATLAB R2021b to compare the first script segment of the human voice against a recording of the same segment produced by neural TTS voices from three vendors commonly used for VHA interventions: Amazon Polly, Microsoft Speech, and Voicemaker Pro. In total, 37 neural voices with a male timbre were compared to the human voice. Each synthesized voice was saved as a 44.1 kHz, 16-bit wave audio file.

Voices were evaluated on three dimensions of vocal prosody: rate of speech (excluding pauses), fundamental frequency (pitch), and pitch variation. To find the TTS voice that most closely matched the human voice, we calculated a total difference score by summing the differences across these three features, giving less weight (10%) to pitch variation, as pitch differences occur at every sample and can otherwise dominate the overall score. The Voicemaker John neural voice had the smallest overall difference, making it the closest match to the human voice.

##### Altered Text-to-speech Voice Condition.

We used the same neural TTS voice selected for the text-to-speech voice condition to generate a new voice designed to portray an increased level of expertise through vocal confidence using speech synthesis markup language (SSML). SSML is an XML-based language used to alter vocal features of a TTS voice during generation. Using SSML, we increased the rate of speech by 10% [14, 38] to a maximum of 191 words per minute [14], lowered the pitch (fundamental frequency) by 20% [13, 14, 18], and added a falling pitch contour [18] to produce the altered TTS voice. The voice was generated as a wave audio file with a sample rate of 44.1 kHz and a 16-bit sample size.

#### 3.1.2 Virtual Health Assistant.

The model and animations for the White male VHA (see Figure 1) were acquired from prior VHA studies on promoting colorectal cancer screening [43]. Lip-sync animations were created using FaceFX Professional 2017.1. The VHA intervention consisted of four segments rendered to video in Unity 2019.3.12f1 at 30 frames per second with a resolution of 1080x954 and 2-channel (stereo) audio at a 44.1 kHz sample rate.

#### 3.1.3 Doctor Introduction.

A video of a human medical doctor provided a brief explanation of the VHA and healthcare intervention to participants prior to the VHA intervention. The doctor’s introduction video had a video resolution of 1080x954 pixels and 2-channel audio (sample rate of 44.1 kHz), which was 41 seconds long.

### 3.2 Methodology

#### 3.2.1 Study Design.

This study investigated the impact of TTS voices on intentions to screen for CRC. We conducted a 3-condition between-participants study where we manipulated the voice used by the VHA (human vs. TTS vs. altered TTS). Participants in each condition were presented with information on the risks of CRC and instructions on the ease of using a fecal immunochemical test (FIT). Participants were randomly and evenly assigned to one of three conditions, each featuring the same White male virtual health assistant differing only in voice type.

#### 3.2.2 Procedure.

Participants were pre-screened as part of the Qualtrics Panel recruitment process to ensure they met the eligibility requirements for the study (see Section 3.2.5 for eligibility requirements). After consenting, participants were randomized to one of the three voice conditions (human actor vs. TTS vs. altered TTS).

Participants were shown a 40-second video of a white male medical doctor who provided an overview of the VHA. Participants were then presented with the healthcare intervention led by the VHA, which consisted of approximately 6.5 minutes of information on the importance of screening for CRC, an overview of CRC screening methods, the convenience of using a FIT home stool test kit, and a brief overview of how to use the FIT home stool test kit. Participants then evaluated the effectiveness of the intervention in a series of survey questions, including attention and manipulation checks. Participants were asked to provide demographic information about themselves and were thanked for participating.

#### 3.2.3 Measures.

Following the digital healthcare intervention, participants reported on their intentions to get screened for CRC. We used three 5-point Likert questions modified from prior literature on CRC screening intentions[6]. Table 1 lists the screening intention questions and response options.

#### 3.2.4 Manipulation Check.

To assess the effectiveness of our voice manipulation in the study, participants were asked to rate the accuracy of the information provided by the VHA. This rating was based on a 5-point Likert scale, where 1 represents strongly disagree, and 5 represents strongly agree with the statement, “The information provided by the VHA was accurate.” The results showed that the TTS voice had a mean perceived accuracy rating of 4.05 (SD = 1.10), while the altered TTS voice had a slightly higher mean of 4.19 (SD = 1.07). The difference in perceived accuracy between these two voice conditions was statistically significant, as indicated by the ART-ANOVA result with a p-value of 0.0177. The finding of differences in perceived accuracy highlights the role that vocal alterations play in the perception of content validity. This suggests that manipulating the voice to increase expertise and vocal confidence successfully influenced participants’ perceptions of accuracy–an indicator of confidence[19] and expertise[28]–affirming our manipulation’s effectiveness.

#### 3.2.5 Participants.

A total of 313 participants were recruited using a Qualtrics Panel for this Institutional Review Board (IRB) approved study. Participants were pre-screened to include those who self-described as Black or African American adults between 45 and 75 years of age and who were currently out of guidelines for CRC screening. Colorectal cancer screening is recommended for adults between 45 and 75. Current guidelines for CRC screening include colonoscopy every 10 years, sigmoidoscopy every five years, Cologuard DNA screening every three years, or home stool test every 12 months. Data from 15 participants were excluded from the analysis due to an error in the initial study logic flow. Participants’ reported age range was from 45 to 74, with a mean age of 52.5 and a median age of 50. Participants reported their gender as female (n = 148) and male (n = 150). Eight participants reported being Hispanic or Latino.

#### 3.2.6 Data Analysis.

Data analysis for this study was conducted using R script version 4.3.2 within the RStudio environment version 2023.06.1. Testing for significant differences used an Aligned Rank Transform (ART) procedure to prepare our nonparametric data for parametric testing [40]. This method facilitated a detailed evaluation of responses to 5-point Likert-scale questions using an analysis of variance (ANOVA). Equivalence testing was conducted using the Two One-Sided Tests (TOST) procedure implemented with a Wilcoxon rank-sum-based approach, suitable for ordinal, non-normally distributed data. We used an equivalence bound of ±0.20, which is more conservative than the ±0.30 bound recommended by Lakens for 5-point Likert-type scales [22].

### 3.3 Results

#### 3.3.1 General Intent.

Responses to “I want to get screened for colon cancer” were analyzed between the *human voice* (n = 106), *text-to-speech voice* (n = 106), and *altered text-to-speech voice* (n = 86) conditions. An ART-ANOVA showed no significant differences between conditions (F(2, 295) = 2.564, p = 0.079). A nonparametric equivalence test using the Wilcoxon-based TOST procedure was conducted to compare General Intent between the human and TTS voices. At an alpha level of 0.05 and with equivalence bounds of ±0.20, the test indicated statistical equivalence (WMW = 6742, p = 0.005). The estimated location shift was effectively zero (90% CI: [−0.00005, −0.00002]), well within the equivalence bounds. The rank-biserial correlation was −0.13 (90% CI: [−0.26, −0.00]), indicating a small effect size.

#### 3.3.2 Provider Intent.

Participants’ responses to “I will talk to my healthcare provider about colon cancer screening” were analyzed between the *human voice* (n = 106), *text-to-speech voice* (n = 106), and *altered text-to-speech voice* (n = 86) conditions. ART-ANOVA showed no significant difference between the voice conditions (F(2, 295) = 0.0652, p = 0.937). A nonparametric equivalence test using the Wilcoxon-based TOST procedure was conducted to compare Provider Intent between the human and TTS voices. At an alpha level of 0.05 and with equivalence bounds of ±0.20, the test indicated statistical equivalence (WMW = 7436, p < 0.001). The estimated location shift was effectively zero (90% CI: [−0.00004, 0.00005]), well within the equivalence bounds. The rank-biserial correlation was −0.02 (90% CI: [−0.15, 0.11]), indicating a negligible effect size.

#### 3.3.3 FIT Intent.

Participants’ responses to “I will talk to my healthcare provider about colon cancer screening with FIT” were analyzed between the *human voice* (n = 106), *text-to-speech voice* (n = 106), and *altered text-to-speech voice* (n = 86) conditions. ART-ANOVA showed no significant difference between the voice conditions (F(2, 295) = 0.4391, p = 0.645). A nonparametric equivalence test using the Wilcoxon-based TOST procedure was conducted to compare FIT Intent between the human and TTS voices. At an alpha level of 0.05 and with equivalence bounds of ±0.20, the test indicated statistical equivalence (WMW = 7091, p < 0.001). The estimated location shift was effectively zero (90% CI: [−0.00004, 0.00006]), well within the equivalence bounds. The rank-biserial correlation was −0.07 (90% CI: [−0.20, 0.06]), indicating a negligible effect size.

### 3.4 Investigating the Effects of Voice Modality Discussion

Study 1 evaluated how voice type affects participants’ intentions to screen for CRC in a VHA-led intervention. Specifically, it asked: **how does closely matching a TTS voice’s pitch, rate of speech, and pitch variation to a human voice affect participant intention to screen for CRC in a VHA-led healthcare intervention?** Two hypotheses were tested: H1 predicted that a prosodically matched TTS voice would produce screening intentions equivalent to a human voice. H2 predicted that a TTS voice altered to sound more expert and confident would lead to higher screening intentions than the other conditions.

Results provided support for H1. There were no statistically significant differences between the human voice and matched text-to-speech voice conditions across the three intent measures (general intent, provider intent, and FIT-specific intent), equivalence testing confirmed that participant responses were statistically equivalent within the predefined bounds. These findings indicate that–when matched on key prosodic features–a TTS voice can be as effective as a human voice in influencing health-related behavioral intentions.

However, H2 was not supported. The altered TTS voice–designed to sound more authoritative–did not produce significantly higher intent scores than the matched TTS or human voice conditions. This suggests that the specific vocal enhancements employed (e.g., adjusted pitch range, rate of speech) were not sufficient to meaningfully increase perceived credibility or motivation to act. It is also possible that the overall effectiveness of the VHA intervention content limited the room for additional persuasive impact, leading to a ceiling effect in intent responses.

These findings hold important implications for the design of VHA-led interventions. Support for H1 demonstrates that well-matched TTS voices can function as viable replacements for human-recorded voices, offering comparable behavioral outcomes while also affording critical advantages in scalability and adaptability[41]. In contrast, the lack of support for H2 suggests that increasing vocal authority through prosodic manipulation alone may not be sufficient to enhance effectiveness and that other factors may be necessary to produce stronger effects.

Overall, Study 1 provides evidence that carefully matched TTS voices are not only effective but essential for the development of adaptable, culturally responsive health interventions. Their adaptability makes them uniquely suited to environments where tailoring the content and delivery of content in real-time is necessary to reach underserved populations.

## 4 User Preference Study: Understanding Participants’ Choice

We ran a user preference study to understand some of the racial / ethnographic preferences of the virtual health assistant older Black or African American adults have when taking part in a digital healthcare intervention designed to promote CRC screening.

### 4.1 System Description

In the user preference study, participants were given choice options that controlled how the information on CRC was presented to participants (see Section 4.2.1 for Study Design).

#### 4.1.1 Virtual Health Assistants.

The user preference study used four images of VHA models participants could choose from to provide information on the importance of screening for CRC: Black Female, Black Male, White Female, and White Male (Figure 1).

#### 4.1.2 Doctor Introduction.

The choice to include an introduction by a human medical doctor presented participants with a video that provided a brief explanation of the VHA and healthcare intervention. The medical doctor is matched on race and gender to the VHA that the participants selected.

### 4.2 Methodology

#### 4.2.1 Study Design.

In our user preference study, participants were given the following choice options for the healthcare intervention: (1) VHA model (Black male, Black female, White male, White female), (2) Intervention modality (interactive VHA or text-based), and (3) Inclusion of an introduction to the VHA and intervention by a human doctor (yes or no). Participants were presented with the same information on CRC screening as in the main study. Figure 1 depicts the four VHA models.

#### 4.2.2 Procedure.

Participants were pre-screened as part of the Qualtrics Panel recruitment process to ensure they met the eligibility requirements for the study (see Section 3.2.5 for eligibility requirements). After consenting, participants were asked to choose from the options for the VHA intervention: VHA model (Black male, Black Female, White male, or White female), intervention modality (text or video), and inclusion of a human doctor introduction (yes or no). Participants were asked to provide demographic information about themselves and were thanked for participating.

#### 4.2.3 Participants.

A total of 317 participants were recruited using a Qualtrics Panel for this Institutional Review Board (IRB) approved study. Participants underwent the same prescreening as the main study. Participants’ reported age range was from 45 to 74, with a mean age of 52.4 and a median age of 50. Participants reported their gender as female (n = 159) and male (n = 158).

#### 4.2.4 Data Analysis.

A Pearson’s Chi-squared test was used to analyze participant choice options, with Fisher’s Exact Test with Bonferroni adjustment used for pairwise comparisons.

### 4.3 Results

Participants (n = 317) in the user preference study were asked to select from the following options for their healthcare intervention: VHA model(race and gender), intervention modality (text-based or interactive video), and with or without human doctor introduction.

#### 4.3.1 Virtual Health Assistant Model.

Participants chose the VHA option of Black Female at 58.04% (n = 184), Black Male at 30.28% (n = 96), White Female at 7.57% (n = 24), and White Male at 4.1% (n = 13). A chi-squared test revealed that the distribution of the VHA chosen was statistically significant, *X*^2^ = 235.9, df = 3, p < 0.001. Figure 2 shows the distribution of VHA choice selection and pairwise comparison of significant differences.

When examining the covariate of participant gender for the choice of VHA, 84.28% of female participants (n = 134) chose Black Female, 7.55% of female participants (n = 12) chose Black Male, 5.66% of female participants (n = 9) chose White Female, and 2.52% of female participants (n = 4) chose White Male compared to 31.65% of male participants (n = 50) chose Black Female, 53.16% of male participants (n = 84) chose Black Male, 9.49% of male participants (n = 15) chose White Female, and 5.7% of male participants (n = 9) chose White Male. This difference in VHA choice by gender was statistically significant, *X*^2^ = 95.77, df = 3, p < 0.001.

#### 4.3.2 Intervention Modality.

Participants chose the modality of interactive VHA at 50.47% (n = 160) and text-only at 49.53% (n = 157). A chi-squared test revealed that this distribution of intervention modality chosen was not statistically significant, *X*^2^ = 0.03, df = 1, p = 0.8662.

#### 4.3.3 Human Doctor Introduction.

Participants chose the doctor introduction at 64.04% (n = 203) versus no doctor introduction at 35.96% (n = 114). A chi-squared test revealed that this distribution of doctor introduction chosen was statistically significant, *X*^2^ = 24.99, df = 1, p < 0.001.

### 4.4 User Preference Discussion

The user preference study addressed the research question: **what are Black or African American adults’ preferences regarding VHA characteristics, modality, and physician involvement in a VHA-led CRC screening intervention?** By allowing participants to select the race and gender of the VHA, the intervention modality (interactive video or text), and whether to include a human doctor introduction, the study offered insight into key design considerations for culturally responsive health communication.

Findings revealed a strong preference for the Black female VHA model, particularly among female participants. This result provides support in parallel to previous research demonstrating a preference for racial matching of medical professionals[23] and a preference for female virtual agents in mental health interventions[11]. While preferences for delivery modality were evenly split between interactive video and text-based formats, a clear majority favored including a brief introduction from a human doctor, suggesting that even minimal human involvement may help establish credibility.

Taken together, these results emphasize the need for intervention flexibility. Systems must be designed to dynamically adapt VHA characteristics and delivery methods to meet individual needs. Such adaptability is only feasible through the use of computer-generated content, reinforcing the broader argument for their necessity in scalable digital health interventions.

## 5 Study 2: The Influence of Voice Modality Based on Choice

In the user preference study, we identified the Black Female VHA as the preferred choice of VHA models by participants. Building off this finding, our second study examined how a TTS voice closely matched a human voice influences participant intentions to screen for CRC in a healthcare intervention led by a Black Female VHA.

### 5.1 System Description

In our second study, we employed two different voice conditions to examine the impact of TTS voices on intentions to screen for CRC: human voice and text-to-speech voice conditions (see Section 5.2.1 for Study Desig).

#### 5.1.1 Human Voice Condition.

The human voice in Study 2 was selected in the same manner as Study 1 (see Section 3.1.1).

#### 5.1.2 Text-to-speech Voice Condition.

The TTS voice in Study 2 was selected using the same measures as Study 1 among voices from the same 3 vendors (see Section 3.1.1). In all, we compared 45 neural voices with a female timbre to the human voice. The Voicemaker Scarlet neural voice achieved the lowest total difference score, indicating the closest match to the human voice across the three measured dimensions.

#### 5.1.3 Virtual Health Assistant.

We used the Black female VHA from the user preferences study (see Figure 1) in our second study. The VHA was displayed to participants in the same method as in Study 1 (see Section 3.1.2).

### 5.2 Methodology

#### 5.2.1 Study Design.

To further explore the impact of TTS voices on intentions to screen for CRC, we employed a between-participants study where we manipulated the voice used by the VHA (human vs. text-to-speech). Participants in each condition were presented with information by a Black Female VHA on the risks of CRC and instructions on the ease of using a fecal immunochemical test (FIT).

#### 5.2.2 Procedure.

Study 2 used the same procedure as Study 1 without the introduction video by a human Doctor (see Section 3.2.2).

#### 5.2.3 Measures.

Study 2 used the same Likert scale measures on screening intentions as Study 1 (see Section 3.2.3 and Table 1).

#### 5.2.4 Participants.

A total of 106 participants were recruited using Prolific for this Institutional Review Board (IRB) approved study. Participants were pre-screened to include those who self-described as Black or African American adults between 45 and 75 years of age and who were currently out of guidelines for CRC screening. Participants’ reported age range was from 45 to 74, with a mean age of 52.2 and a median age of 50. Participants reported their gender as female (n = 71) and male (n = 35).

#### 5.2.5 Data Analysis.

Data analysis for this study was conducted in a manner similar to Study 1 (see Section 3.2.6).

### 5.3 Results

#### 5.3.1 General Intent.

Responses to “I want to get screened for colon cancer” were analyzed between the *human voice* (n = 53) and *text-to-speech voice* (n = 53) conditions. An ART-ANOVA showed no significant difference in response between conditions (F(1, 104) = 0.8784, p = 0.351). A nonparametric equivalence test using the Wilcoxon-based TOST procedure was conducted to compare General Intent between the human and TTS voices. At an alpha level of 0.05 and with equivalence bounds of ±0.20, the test indicated statistical equivalence (WMW = 1663, p = 0.049). The estimated location shift was effectively zero (90% CI: [−0.00003, 0.00003]), well within the equivalence bounds. The rank-biserial correlation was −0.10 (90% CI: [−0.28, 0.08]), indicating negligible effect size.

#### 5.3.2 Provider Intent.

Participants’ responses to “I will talk to my healthcare provider about colon cancer screening” were analyzed between the *human voice* (n = 53) and *text-to-speech voice* (n = 53) conditions. ART-ANOVA showed no significant difference between the voice conditions (F(1, 104) = 0.0652, p = 0.233). A nonparametric equivalence test using the Wilcoxon-based TOST procedure was conducted to compare Provider Intent between the human and TTS voices. At an alpha level of 0.05 and with equivalence bounds of ±0.20, the test indicated statistical equivalence (WMW = 1717, p = 0.022). The estimated location shift was effectively zero (90% CI: [−0.00003, 0.00002]), well within the equivalence bounds. The rank-biserial correlation was −0.12 (90% CI: [−0.30, 0.06]), indicating a negligible effect size.

#### 5.3.3 FIT Intent.

Participants’ responses to “I will talk to my healthcare provider about colon cancer screening with FIT” were analyzed between the *human voice* (n = 53) and *text-to-speech voice* (n = 53) conditions. ART-ANOVA showed no significant difference between the voice conditions (F(1, 104) = 0.7200, p = 0.398). A nonparametric equivalence test using the Wilcoxon-based TOST procedure was conducted to compare FIT Intent between the human and TTS voices. At an alpha level of 0.05 and with equivalence bounds of ±0.20, the test indicated statistical equivalence (WMW = 1803, p = 0.005). The estimated location shift was effectively zero (90% CI: [−0.00001, 0.00003]), well within the equivalence bounds. The rank-biserial correlation was −0.09 (90% CI: [−0.27, 0.10]), indicating a negligible effect size.

### 5.4 The Influence of Voice Modality Based on Choice Discussion

Study 2 served as a replication of the first hypothesis from Study 1 (see H1 in Section 1), which predicted that a TTS voice, when matched to a human voice on pitch, speech rate, and pitch variation, would elicit equivalent behavioral intentions to screen for CRC. This study included a new participant sample and tested only the human and matched TTS voice conditions.

The findings were consistent with those of Study 1. No statistically significant differences were observed across general intent, provider intent, or FIT-specific intent measures. Equivalence testing confirmed that participant responses to the TTS voice were statistically equivalent to those for the human voice across all metrics. By replicating the original result, Study 2 strengthens the evidence that prosodically matched TTS voices can serve as effective substitutes for human voices in VHA-led health interventions. These findings further support using TTS as a viable foundation for adaptable, scalable, and culturally responsive health communication.

## 6 Overall Discussion

Across two studies, we examined the role of TTS voices in VHA-led healthcare interventions, specifically focusing on CRC screening intentions among Black or African American adults. The results of these studies offer evidence that TTS voices – when matched on key prosodic features such as pitch, rate of speech, and pitch variation – can produce behavioral intentions equivalent to those elicited by human voices.

In Study 1, we found no significant differences between human and TTS voice conditions across general intent, provider intent, and FIT-specific intent measures. More importantly, equivalence testing confirmed that responses to the matched TTS voice were statistically equivalent to those elicited by a human voice. A third condition, which altered the TTS voice to increase expertise and vocal confidence, did not show a measurable improvement in screening intent, suggesting that subtle manipulations to vocal tone alone may not be sufficient to enhance perceived credibility or persuasiveness within this context.

Study 2 replicated these findings with a new participant sample and a streamlined voice manipulation (human vs. matched TTS voice only). Once again, no significant differences emerged between conditions, and equivalence was confirmed across all intention outcomes. This replication supports the robustness of the original finding and further underscores the viability of using matched TTS voices in health-focused behavioral interventions.

The user preference study examined user preferences in the design of VHA-led interventions. Participants overwhelmingly selected VHAs reflecting their racial and gender identity, with a strong preference for the Black female VHA model. While preferences for delivery modality (interactive video vs. text-based) were evenly split, most participants favored interventions that began with a brief introduction from a human doctor. These findings reinforce the importance of representation and trust-building in digital health design and highlight the need for flexible systems capable of dynamically adjusting VHA characteristics and content.

Taken together, the results across this multi-study strongly support the use of prosodically matched TTS voices in VHA-led interventions. More critically, these findings emphasize the necessity of TTS voices for achieving intervention adaptability – particularly when designing culturally responsive systems that serve underserved populations. Pre-recorded human voices, while natural-sounding, lack the flexibility needed for real-time personalization. TTS enables dynamic message tailoring based on user characteristics and preferences, making it an essential component of scalable and equitable digital health interventions.

## 7 Context and Limitations

This multi-study was conducted online using Qualtrics, with participants accessing it from their personal devices at their convenience. Consequently, factors like environmental distractions, screen size, resolution, and audio quality could not be controlled and may have influenced participants’ perceptions of the intervention or the VHA. However, enabling access anytime and anywhere offers practical benefits that outweigh minor variations in intervention quality.

While our study was limited to participant screening intentions, the correlation to actual behavior in colorectal cancer screenings is significant. Chambers et al. examined screening intentions against behavior and found that 70.2% of those who indicated a strong intention to screen went on to complete the screening [5], highlighting the importance of increasing screening intentions.

The primary aim of this multi-study was to examine the use of TTS voices as an equivalent alternative to human voices for use in a healthcare intervention led by a VHA with participants from an underserved population. As this was a very specific population (see Section 3.2.5), the generalizability of these findings is limited. Additionally, only two TTS to human voice equivalency comparisons were made to examine intentions to screen for CRC. This further limits the generalizability of the findings in these studies.

## 8 Conclusion and Future Work

This research investigated the use of prosodically matched TTS voices in VHA-led interventions to determine whether such voices can elicit behavioral intentions equivalent to those of human voices. Across two studies, we found consistent evidence supporting the equivalence of matched TTS and human voices in influencing participant intent to screen for CRC. Our user preference study highlighted participant preferences regarding VHA model characteristics, modality, and human involvement, underscoring the importance of designing interventions that are culturally responsive and adaptable. This work contributes to the growing literature on virtual human design by providing support for using adaptable TTS as a foundation for scalable digital healthcare systems.

Given the non-significant effects of the altered voice in Study 1, future research should refine voice modulation techniques to better align prosodic features to influence engagement and trust. Exploring how voice naturalness interacts with VHA visual fidelity is also critical, as voice-appearance mismatches could reduce trust in photorealistic agents [7, 15]. Future studies should extend beyond Black or African American populations as well as focus on actual behavior, such as confirmed CRC screenings. Finally, controlled experiments are needed to determine whether personalized VHA configurations causally impact health outcomes, informing the design of more effective, culturally responsive interventions.

## Supplementary Material

Supplementary Video

## Figures and Tables

**Figure 1:**
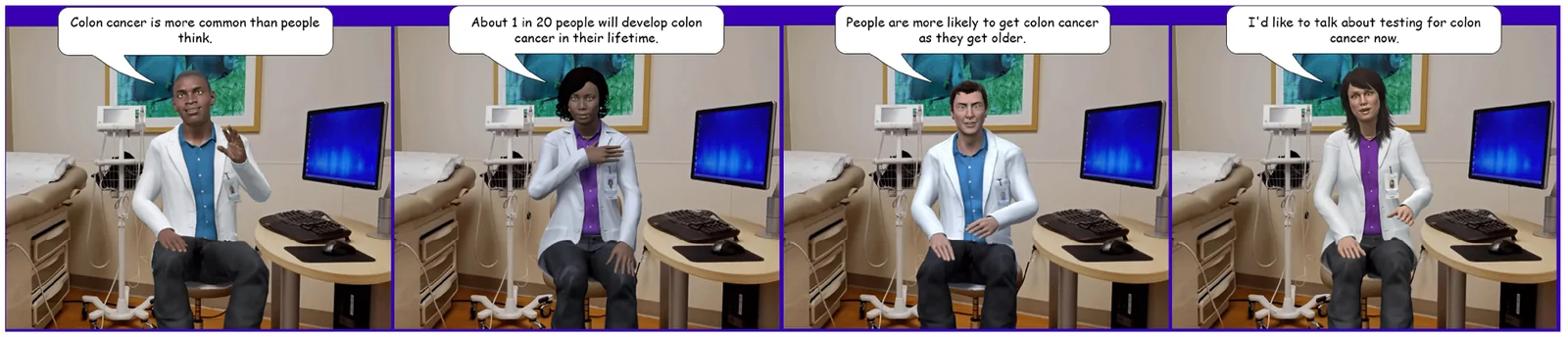
Representation of virtual health assistant models

**Figure 2:**
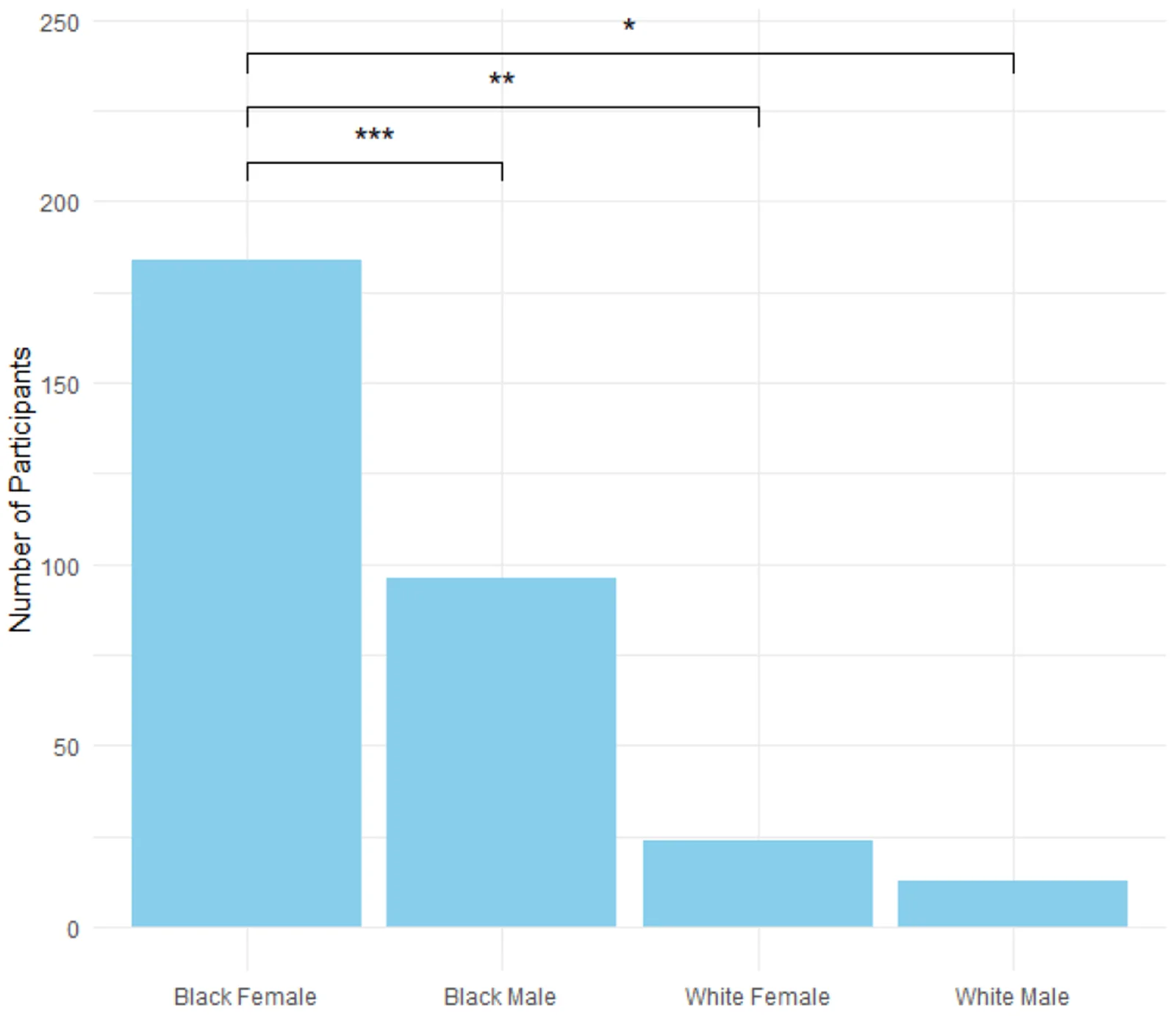
Bar plot showing the number of participants who selected each VHA model, categorized by VHA race and gender. Significance: *p < .05, **p < .01, ***p < .001

**Table 1:** Summary of Measures Used to Assess Screening Intentions

Measure	Question	Scale
General Intent	I want to get screened for colon cancer	Strongly Disagree (1) to Strongly Agree (5)
Provider Intent	I will talk to my healthcare provider about colon cancer screening	Strongly Disagree (1) to Strongly Agree (5)
FIT Intent	I will talk to my healthcare provider about colon cancer screening with FIT	Strongly Disagree (1) to Strongly Agree (5)

## CCS Concepts

• **Human-centered computing** → **Human computer interaction (HCI)**; *Interaction design*; • **Applied computing** → *Consumer health*.
